# Cytotoxicity Studies of the Crude venom and Fractions of Persian Gulf Snail (*Conus textile*) on Chronic Lymphocytic Leukemia and Normal Lymphocytes

**DOI:** 10.31557/APJCP.2021.22.5.1523

**Published:** 2021-05

**Authors:** Ahmad Salimi, Shayan Salehian, Akram Aboutorabi, Amir Vazirizadeh, Vahed Adhami, Seyyed Hossein Sajjadi Alehashem, Enayatollah Seydi, Jalal Pourahmad

**Affiliations:** 1 *Traditional Medicine and Hydrotherapy Research Center, Ardabil University of Medical Sciences, Ardabil, Iran. *; 2 *Department of Pharmacology and Toxicology, School of Pharmacy, Ardabil University of Medical Sciences, Ardabil, Iran. *; 3 *Department of Pharmacology and Toxicology, Faculty of Pharmacy, Shahid Beheshti University of Medical Sciences, Tehran, Iran. *; 4 *Halal Research Center of Islamic Republic of Iran, Tehran, Iran. *; 5 *Persian Gulf Research Institute, Marine Biology and Fishery Sciences Department, Persian Gulf University, Iran. *; 6 *Department of Occupational Health and Safety Engineering, School of Health, Alborz University of Medical Sciences, Karaj, Iran. *; 7 *Research Center for Health, Safety and Environment, Alborz University of Medical Sciences, Karaj, Iran.*

**Keywords:** Conus textile, B-Lymphocyte, mitochondria, apoptosis- oxidative stress, Persian Gulf

## Abstract

**Background:**

Marine animals have been considered by many researchers due to their various pharmacological effects. One group of marine animals that have been studied is cone snails. The conotoxin obtained from these marine animals has various therapeutic effects.

**Methods:**

This study was designed to investigate the apoptotic effects of crude venom of Conus textile and its fractions (A and B) on chronic lymphocytic leukemia (CLL) cells. Accordingly, parameters such as cell viability, reactive oxygen species (ROS) level, collapse in mitochondrial membrane potential (MMP), lysosomal membrane damage and caspase-3 activation were evaluated.

**Results:**

The results showed that the crude venom (50, 100 and 200 µg/ml) from Conus textile and its fraction B (50, 100 and 200 µg/ml) significantly reduced viability in CLL B-lymphocyte. In addition, exposure of CLL B-lymphocyte to fraction B (50, 100 and 200 µg/ml) was associated with an increase in the level of ROS, the collapse of the MMP, damage to the lysosomal membrane, and activation of caspase-3.

**Conclusion:**

According to results, it was concluded that fraction B from crude venom of Conus textile causes selective toxicity on CLL B-lymphocyte with almost no effect on a normal lymphocyte. Furthermore, this venom fraction could be a promising candidate for induction of apoptosis in patients with CLL through the mitochondrial pathway.

## Introduction

Today, cancer is one of the most important health problems and the cause of human mortality in worldwide. According to the World Health Organization in 2018, cancer is responsible for the death of 9.6 million humans worldwide (Oroz-Parra et al., 2020; Sun et al., 2020). Chronic lymphocytic leukemia (CLL) is the most common type of leukemia that occurs in adults. CLL cannot be treated with traditional therapies and defects in signaling apoptosis are involved in the progression of the malignancy (Salimi et al., 2015; Teoh and Goh, 2021). Natural compounds of marine origin have been considered by many researchers as one of the important sources for drug discovery. In recent years, many of these compounds have been used in the treatment of malignancies due to their various pharmacological properties. Furthermore, animal venoms (such as cone snails) have been suggested as an alternative approach for anti-cancer therapies. Animal venoms have different pharmacological functions due to their unique bioactive molecules. Isolation of compounds with specific anticancer effects from animal venoms has become an exciting topic in cancer research (Abdel-Rahman et al., 2013; Ruiz-Torres et al., 2017; Chatterjee, 2018; Wali et al., 2019; Jimenez et al., 2020; Sukmarini, 2021).

Marine cone snails (Conus) are a large group of carnivorous predators that are distributed in tropical and subtropical water. Research has shown that there are more than 700 species of Conus around the world. Venoms isolated from cone snails have various pharmacological effects (such as anti-cancer effect). Furthermore, the venom of these marine animals contains a rich source of a peptide called conotoxins. Conotoxins have been reported to have several pharmacological and physiological activities (Gao et al., 2017a; Gao et al., 2017b; Fu et al., 2018; Alburae and Mohammed, 2020; Oroz-Parra et al., 2020). 

In recent years, mitochondria have been considered by many researchers as a therapeutic target. This organelle is involved in the generation of reactive oxygen species (ROS) and energy and in signaling cell death. Research has shown that cancer cells are vulnerable to a rise in ROS generation and subsequent oxidative stress. Also, a rise in the level of ROS can be associated with the induction of cell death signaling. Therefore, an increase in the level of ROS can kill cancer cells (Seydi et al., 2016; Seydi et al., 2018). Our previous study showed that crude venom of Conus textile has the ability to increase ROS and induction of apoptosis signaling in U87MG human glioma cells through the mitochondrial pathway (Salimi et al., 2020). This research was aimed to investigate the apoptotic effects of crude venom of Conus textile and its fractions (A and B) on CLL.

## Materials and Methods

*Chemicals*

2, 7-dicloroflurescein diacetate (DCHF-DA), rhodamine 123 (Rh 123) and acridine orange were purchased from Sigma-Aldrich Chemical Co. (St. Louis, MO, USA). Furthermore, caspase-3 assay kit were purchased from Sigma-Aldrich Chemical Co. (Taufkirchen, Germany). All other chemicals were of the highest commercial grade available.

*Collection and extraction of venom*

The Conus textile samples were collected from Larak Island in the Persian Gulf, in the south of Iran. The stages of toxin extraction are described in our previous study (Salimi et al., 2020).

*Chromatography Methods*

Lyophilized Conus textile venom (100 mg) was dissolved in 2 mL of 0.2 M ammonium acetate buffer. Venom fractions were separated by using a Sephadex G 50 column (2 × 150 cm) previously equilibrated and eluted with 0.2 M ammonium acetate buffer (pH 7.5). And were detected at 280 nm (Rigby et al., 1999). Finally, fractions were lyophilized and kept at 4^o^C for further evaluation. 

*CLL sample and B-lymphocyte isolation*

In this study, samples were collected from 10 patients with CLL. All experiments were performed based on standard protocols approved by the ethic of Shahid Beheshti University of Medical Sciences, Tehran, Iran (ethic number: IR.SBMU.PHARMACY.REC.1398.315). Furthermore, all the CLL patients and healthy donors signed an informed consent form. Subsequently, B- lymphocytes were isolated from the blood of samples (obtained from the CLL patient and healthy donors) using Ficoll-Paque and differential centrifugation (Salimi et al., 2015).

*Cell viability assay*

To perform this test, CLL B- lymphocytes and normal B- lymphocytes were placed in 96-well plates with RPMI 1640 culture medium (104 cells per well). Then, both groups were treated to crude venom and fraction A and B from Conus textile at concentrations of 1, 5, 10, 50, 100 and 200 µg/ml for 12 hours. In the next step, MTT (0.5 mg/ml) was added to both groups for 4 hours. Then, DMSO was added to dissolve the formazone crystals. Finally, the absorbance of the samples was read using a spectrophotometer at 570 nm for cell viability assay (Zhang et al., 2008).

*ROS assay*

At first, CLL B- lymphocytes were placed in 24-well plates with RPMI 1640 culture medium (10^4^ cells per well). Then, CLL B- lymphocytes were treated to fraction B from crude venom of Conus textile (50, 100 and 200 µg/ml). PBS was used to wash CLL B- lymphocytes. In addition, CLL B- lymphocytes were incubated with DCFH-DA reagent and centrifuged for 1 minute at 1,000 rpm. In the following, we removed the DCFH-DA reagent and re-washed the cells with PBS. Finally, fluorescence intensity (DCF) was evaluated using flow cytometry (Cyflow Space-Partec, Germany) and the mean of fluorescence intensity was analyzed by software (FlowJo) (Salimi et al., 2020).

*Mitochondrial Membrane Potential (MMP) collapse assay*

Briefly, CLL B- lymphocytes were placed in 24-well plates with RPMI 1640 culture medium (104 cells per well). Then, CLL B- lymphocytes were treated to fraction B from crude venom of Conus textile (50, 100 and 200 µg/ml). PBS was used to wash CLL B- lymphocytes. In addition, CLL B- lymphocytes were incubated with Rh123 reagent and centrifuged for 1 minute at 1,000 rpm. In the following, we removed the Rh123 reagent and re-washed the cells with PBS. Finally, fluorescence intensity (Rh123) was evaluated using flow cytometry (Cyflow Space-Partec, Germany) and the mean of fluorescence intensity was analyzed by software (FlowJo) (Baracca et al., 2003).

*Evaluation of lysosomal damage*

At first, CLL B- lymphocytes were placed in 24-well plates with RPMI 1640 culture medium (10^4^ cells per well). Then, CLL B- lymphocytes were treated to fraction B from crude venom of Conus textile (50, 100 and 200 µg/ml). PBS was used to wash CLL B- lymphocytes. In addition, CLL B- lymphocytes were incubated with acridine orange reagent and centrifuged for 1 minute at 1,000 rpm. In the following, we removed the acridine orange reagent and re-washed the cells with PBS. Finally, fluorescence intensity (acridine orange) was evaluated using flow cytometry (Cyflow Space-Partec, Germany) and the mean of fluorescence intensity was analyzed by software (FlowJo) (Brunk et al., 1995).

*Caspase-3 activity assay*

Caspase-3 activity was assayed using the Sigma Caspase-3 assay kit (Sigma-Aldrich, Taufkirchen, Germany), and the concentration of the p-nitroaniline released from the substrate at 405 nm used for caspase-3 activity was assayed.

*Statistical analysis*

Results are showed as means ± SD. All statistical analyses were done using the GraphPad Prism software, version 6. All experiments were analyzed using one-way analysis of variance (ANOVA) followed by the Tukey test. Statistical significance was set at P < 0.05.

## Results

*Fractionation of Conus textile toxins*

**Figure 1:**
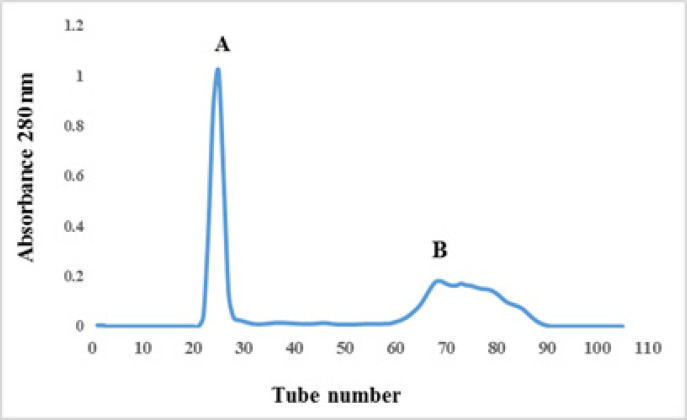
Gel Filtration Diagram of Conus Textile Crude Venom (100 mg), Dissolved Venom in Ammonium Acetate Buffer and Loaded on a Sephadex G50 Column, Previously Equilibrated and Eluted with 0.2 M Ammonium Acetate, pH 7.5, and Detected at 280 nm

In order to obtain active molecules crude venom of Conus textile, was used Sephadex G50 gel filtration. The column was equilibrated and eluted with ammonium acetate buffer (0.2 M). As shown in Figure 1, two main peaks were collected from a Sephadex G-50 column, for further evaluations. 

*Conus textile (crude venom and fraction B) decreased cell viability in CLL B- lymphocytes*

**Figure 2:**
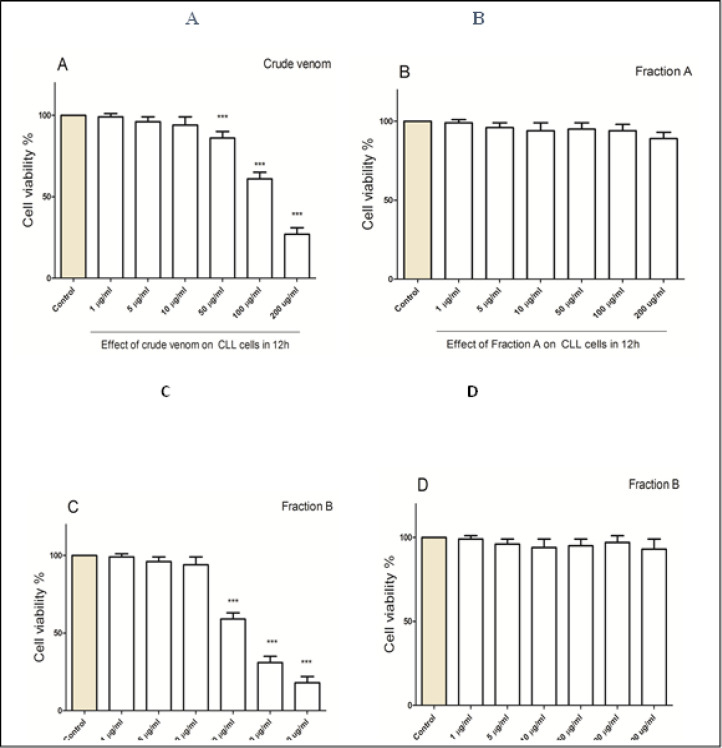
Cell Viability Assay. (A) Effect of crude venom on CLL B- lymphocytes in 12 hours, (B) effect of fraction A on CLL B- lymphocytes in 12 hours, (C) effect of fraction B on CLL B- lymphocytes in 12 hours, (D) effect of fraction B on normal cells in 12 hours. All data were presented as the mean ± S.D (n=3). *** p<0.001 significantly different from the control group

After 12 hours of treatment, the results showed that crude venom of Conus textile at concentrations of 50, 100 and 200 µg/ml decreased cell viability in CLL B-lymphocytes (Figure 2A). Next, the effects of fractions A and B (from crude venom of Conus textile) on cell viability in normal B-lymphocytes and CLL B-lymphocytes were investigated. As shown in Figure 2B, fraction A was not able to reduce cell viability in CLL cells. However, fraction B at concentrations of 50, 100 and 200 µg/ml and after 12 hours of treatment was able to reduce cell viability in CLL B-lymphocytes (Figure 2 C). Furthermore, this effect of fraction B has not been observed on normal B-lymphocytes (Figure 2D). In the following, the effects of fraction B on the desired parameters were investigated.

*Fraction B increased ROS generation in CLL B- lymphocytes*

**Figure 3:**
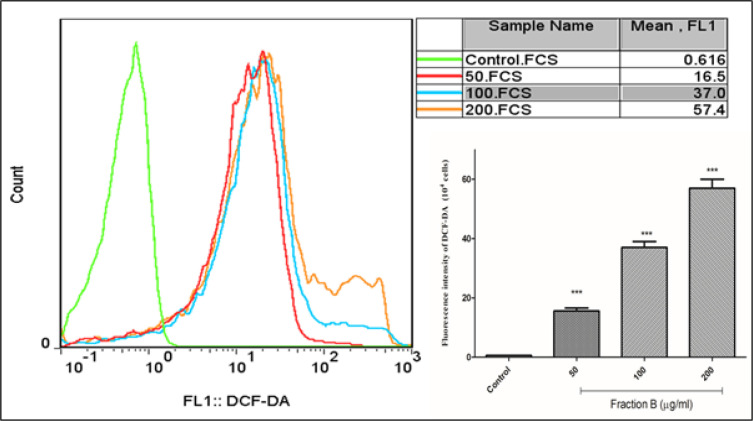
ROS Assay. Effect of fraction B (50, 100 and 200 µg/ml) on ROS generation in CLL B- lymphocytes. All data were presented as the mean ± S.D (n=3). *** p<0.001 significantly different from the control group. DCF-DA: Dichlorofluorescin Diacetate. FCS: Forward Channel Scatter. FL1: Fluorescence Channel 1

To assess the level of ROS generation, CLL B-lymphocytes were treated to different concentrations of fraction B from crude venom of Conus textile. Using flow cytometry, the results showed that fraction B at concentrations of 50, 100 and 200 µg/ml was able to increase the level of ROS in CLL B- lymphocytes (Figure 3). This effect of fraction B has not been observed on normal B-lymphocytes (data not shown.). Compounds that have the ability to increase ROS generation can help kill cancer cells.

*Fraction B induced MMP collapse in CLL B- lymphocytes*

**Figure 4.**
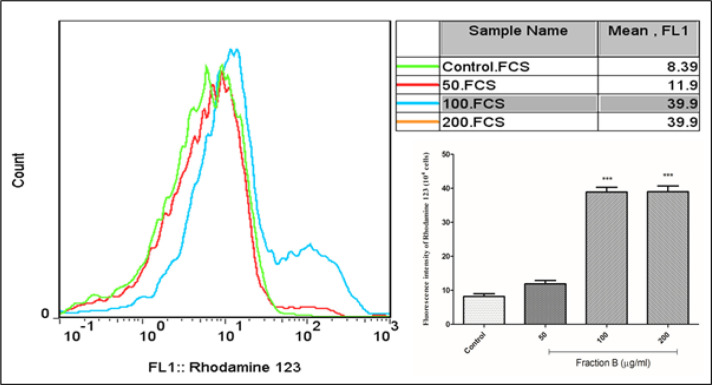
MMP Assay. Effect of fraction B (50, 100 and 200 µg/ml) on MMP collapse in CLL B- lymphocytes. All data were presented as the mean ± S.D (n=3). *** p<0.001 significantly different from the control group. FCS, Forward Channel Scatter; FL1, Fluorescence Channel 1

The collapse in the MMP is considered to be one of the most important events in cell death. Flow cytometry results showed that fraction B at all concentrations (50, 100 and 200 µg/ml) used compared to the control group caused the collapse of the MMP in CLL B-lymphocytes (Figure 4). 

*Fraction B induced lysosomal damage in CLL B- lymphocytes*

**Figure 5:**
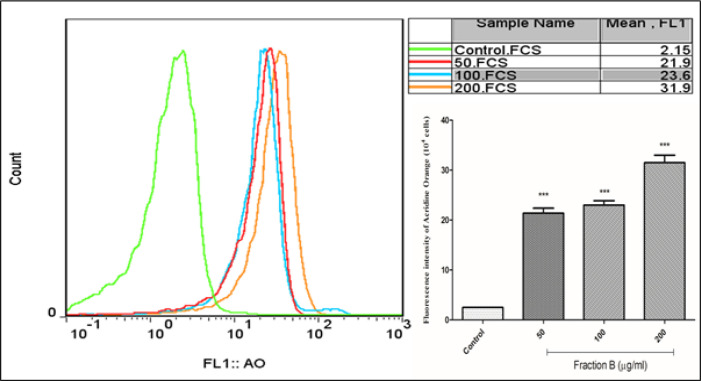
Lysosomal Damage Assay. Effect of fraction B (50, 100 and 200 µg/ml) on lysosomal damage in CLL B- lymphocytes. All data were presented as the mean ± S.D (n=3). *** p<0.001 significantly different from the control group. FCS, Forward Channel Scatter; FL1, Fluorescence Channel 1; AO, Acridine Orange

Flow cytometry results showed that incubation of CLL B-lymphocytes with fraction B at concentrations of 50, 100 and 200 µg/ml significantly (p<0.05) leads to lysosomal damage in the compared to the control group (Figure 5). This effect of fraction B has not been observed on normal cells (data not shown.).

*Fraction B increase caspase-3 activity in CLL B- lymphocytes*

**Figure 6:**
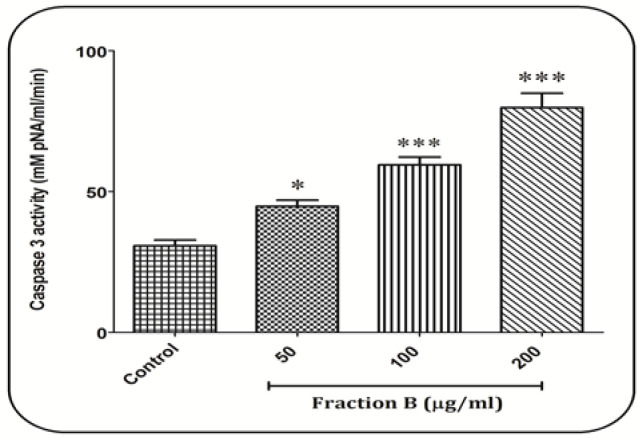
Caspase-3 Activity Assay. Effect of fraction B (50, 100 and 200 µg/ml) on caspae-3 activity in CLL B- lymphocytes. All data were presented as the mean ± S.D (n=3). * p<0.05 and *** p<0.001 significantly different from the control group

In Figure 6, caspase-3 activity in a dose-dependent pattern was significantly increased in CLL B-lymphocytes compared to the control group.

## Discussion

In the present study, the toxicity effects of isolated fractions of Persian Gulf snail crude venom (Conus textile) on CLL B-lymphocytes were investigated. In recent years, according to data from the WHO the number of new cancer cases is on the rise. Cancer treatment is still considered a medical challenge worldwide. Therefore, the discovery of new compounds with anti-cancer effects is essential (Aufschnaiter et al., 2020; Borojeni et al., 2020). Today, venoms isolated from animal sources are of great importance for the treatment of cancer. These venoms contain bioactive compounds such as proteins, salts, peptides and small molecules that have many pharmacological activities. Furthermore, isolated venoms have little toxic effects on normal cells and tissues and also can play a role in the induction of apoptosis signaling (Mirzaei et al., 2020; Roy and Bharadvaja, 2020).

Initially, the results showed that the crude venom and its fraction B had the ability to reduce the viability of CLL B-lymphocytes. These results are in agreement with previous studies that have shown that the use of venoms extracted from marine animals is associated with a decrease in the viability of various cancer cell lines (Catanesi et al., 2021; Rodrigo et al., 2021). Studies have shown that there is an inverse relationship between cell viability and apoptotic signaling (Li et al., 2020; Wang et al., 2020). Therefore, it is possible that the decrease in cell viability by Conus textile in CLL cells is associated with activation of apoptotic signaling. Mitochondria have attracted a lot of attention as a therapeutic target. Mitochondria play important roles in physiological processes including the generation of ROS and apoptosis signaling (Fisher et al., 2020; He et al., 2020). Research has shown that an increased level of mitochondrial ROS in cancer cells is considered a therapeutic target. In addition, studies have shown that the level of free radicals in cancer cells is higher than in normal cells, and an excessive mitochondrial ROS can contribute to the kill of cancer cells. Therefore, compounds that can increase the level of ROS can be used to kill cancer cells.(Seydi et al., 2016; Seydi et al., 2018). 

An association between ROS levels and apoptotic signaling has been reported. ROS can activate apoptotic signaling by releasing pro-apoptotic proteins (such as cytochrome c) from the mitochondria and activation of a caspase cascade (Simon et al., 2000; Li et al., 2003). The results of our study showed that fraction B obtained from Conus textile was able to increase the level of ROS in CLL. These results are in agreement with the results of previous studies that have shown that crude venom of Conus textile and other animal venoms have the ability to increase the level of ROS (Al-Asmari et al., 2018; Salimi et al., 2020). 

The collapse in the MMP is one of the important events in apoptosis that can occur due to an increase in the level of ROS. It has been shown that collapse in the MMP may be associated with the release of pro-apoptotic proteins as well as caspase cascade activation (Seydi et al., 2016; Seydi et al., 2018). The increase in the level of ROS due to fraction B of Conus textile in CLL B-lymphocytes has been associated with the collapse of MMP. These results from this venom have been proven in our previous study on U87MG human glioma cells (Salimi et al., 2020). Lysosomes is considered as another source of ROS and can also play a role in apoptosis signaling (Zhang et al., 2009). The results showed that fraction B in a dose-dependent pattern caused damage to the lysosomal membrane in CLL B-lymphocytes. Apoptosis is a type of cell death and the mitochondrial pathway is one of the most important pathways involved in apoptosis signaling. Caspase 3 plays an important role in apoptosis as a final mediator (Seydi et al., 2016; Zhou et al., 2021). The result showed that fraction B selectively could induce activation of caspase-3 via mitochondrial pathway in CLL B-lymphocytes. These results are in agreement with our previous study (Salimi et al., 2020).

Our findings in this study suggest that fraction B from crude venom of Conus textile has been able to increase the level of ROS in CLL cells by acting on mitochondria and lysosomes. Subsequently, ROS was able to activate caspase-3 by acting on MMP and the release of pro-apoptotic proteins. Accordingly, Conus textile can be a promising source for anticancer drug candidates. 

## Author Contribution Statement

Ahmad Salimi and Enayatollah Seydi contributed to this research in in carrying out the experiments, analyzing the data and writing the paper. Shayan Salehian contributed to this research in carrying out the experiments and performing statistical analysis as the thesis student. Vahed Adhami, Amir Vazirizadeh and Seyyed Hossein Sajjadi Alehashem contributed in this research in carrying out some experiments. Jalal Pourahmad contributed to this research in formulating the research question (s), designing the study, carrying it out as thesis supervisor, analyzing the data, and writing paper. Akram Aboutorabi contributed to this research in designing part of the study and carrying it out as thesis co-supervisor.
